# Vanishing Pituitary Macroadenoma: A Case Report

**DOI:** 10.7759/cureus.838

**Published:** 2016-10-20

**Authors:** Emily P Sieg, Hayk Stepanyan, Russell Payne, Tao Ouyang, Brad E Zacharia

**Affiliations:** 1 Department of Neurosurgery, Penn State Hershey Medical Center; 2 Medical Student, Penn State College of Medicine; 3 Radiology, Penn State Hershey Medical Center

**Keywords:** pituitary macroadenoma, suprasellar mass, adenoma, spontaneous regression

## Abstract

Pituitary macroadenomas are the most common suprasellar lesions in adults and are typically managed surgically through transsphenoidal resection when symptomatic. Due to their close proximity to the optic chiasm, pituitary macroadenomas often present with signs of bitemporal hemianopsia. Alternatively, these tumors can cause mass effect, thus presenting with signs of elevated intracranial pressure or can present with signs and symptoms of endocrine dysfunction. Here, we discuss a 55-year-old male diagnosed with a non-functioning pituitary macroadenoma (NFPA) based on cranial imaging, ophthalmologic exam, and endocrine evaluation. Following diagnosis, the patient was scheduled for transsphenoidal hypophysectomy. On magnetic resonance imaging (MRI) done three and half months later for surgical planning, the tumor had almost completely regressed and only residual pituitary tissue was noted. We describe the presentation and clinical course of the patient, summarize chief differential diagnoses, and discuss potential managements of these conditions.

## Introduction

Pituitary adenomas are common intracranial lesions believed to be present in as many as one in six individuals in recent postmortem radiologic series [1]. Adenomas can be divided into functional and non-functional based on hormonal secretion. Non-functioning pituitary adenomas (NFPA) are the second most common type of pituitary tumor after prolactin-producing tumors. These tumors are further classified as micro or macro based on size, with a 1 cm cutoff typically separating these two categories. Macroadenoma are in close proximity to crucial neural and vascular structures including the pituitary gland, hypothalamus, optic nerve and chiasm and carotid arteries. Left untreated, approximately 50% of macroadenomas will enlarge with more than two-thirds of the patients experiencing worsening of their vision [2]. Given the natural history and lack of efficacy of medical therapy for non-functioning macroadenoma, surgical resection is considered the optimal first line therapy for symptomatic lesions. Diagnostic workup including endocrinologic testing, dedicated sellar or pituitary protocol MRI with dynamic contrast, and ophthalmologic testing are recommended prior to surgical intervention. We present a case of a patient with a macroadenoma and subjective visual disturbance that demonstrated complete radiographic resolution on repeat imaging prior to surgical or medical intervention. Informed consent was obtained from the patient for this study.

## Case presentation

**Figure 1:**
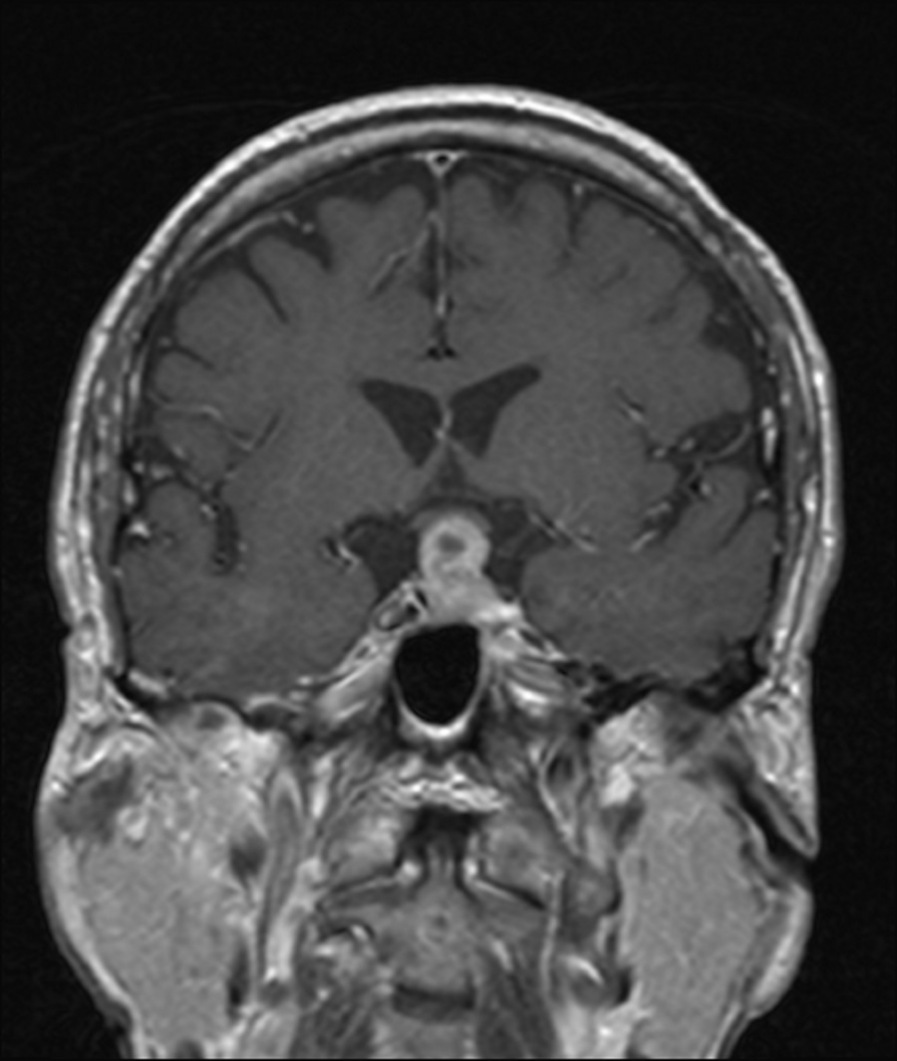
Initial MRI obtained at the time of diagnosis Initial T1 coronal MRI showing a uniformly enhancing sellar mass extending into the suprasellar cistern with mass effect on the optic chiasm.

**Figure 2:**
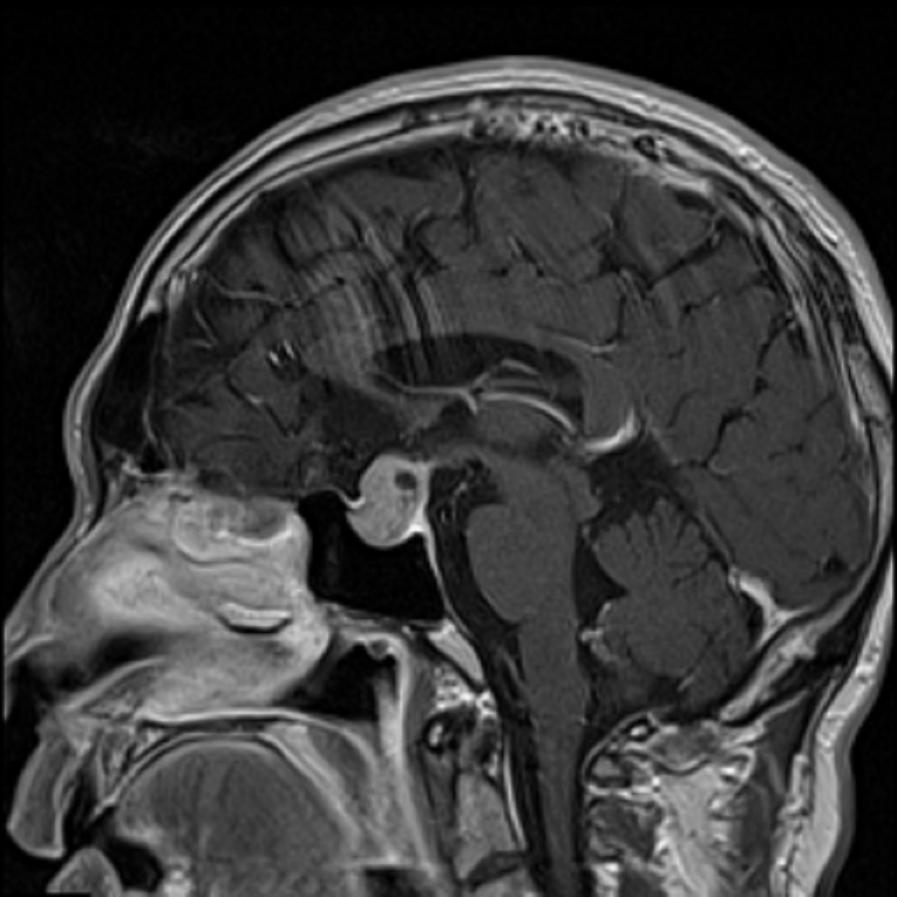
Initial MRI obtained at the time of diagnosis Initial T1 sagittal MRI again showing a sellar mass with a small cystic component in the superior aspect.

The patient initially presented to an outside institution emergency department with severe dyspnea and altered mental status and was found to have systolic heart failure due to a ventricular thrombus. Once stabilized, an MRI of the head revealed a 1.7 x 1.4 x 2.2 cm^3 ^sellar/suprasellar mass (Figures 1-2). The mass was inseparable from normal pituitary tissue and extended into the suprasellar cistern, with mass effect on the optic chiasm. There was a small cystic component in the superior aspect of the mass, which is not uncommonly seen in large adenomas, but otherwise it was uniformly enhancing, without signal characteristics to suggest hemorrhage.

While at the outside institution, a random cortisol level was 5 mcg/dL (normal 3–18). His free T4 was 0.56 ng/dL (normal 0.4–1.64), prolactin was 1.9 ng/mL (normal 2–18), testosterone was 27 ng/dL (normal 270–1070), FSH 6.2 IU/L (normal 1.5–12.4), and LH 1.7 mlU/L (normal 1.9–12.5). 

**Figure 3:**
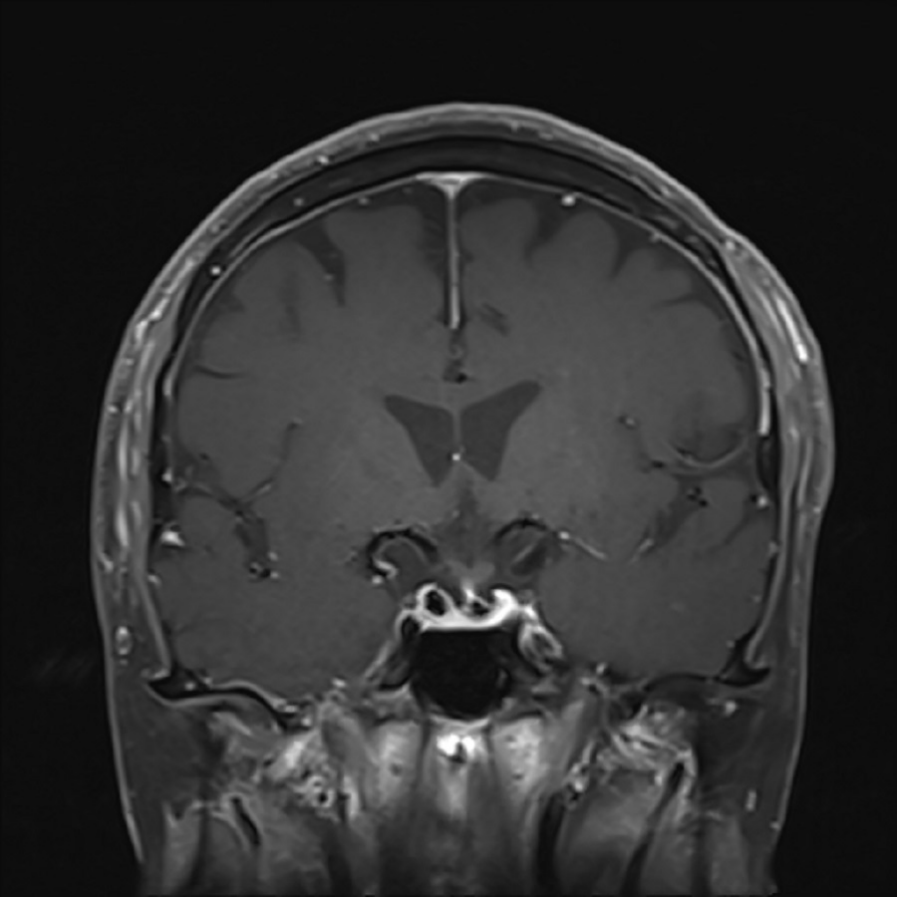
Follow-up MRI obtained approximately three and a half months following initial imaging T1 coronal MRI demonstrating complete resolution of the mass. The infundibulum is now visible and the optic chiasm is in its anatomic position.

**Figure 4:**
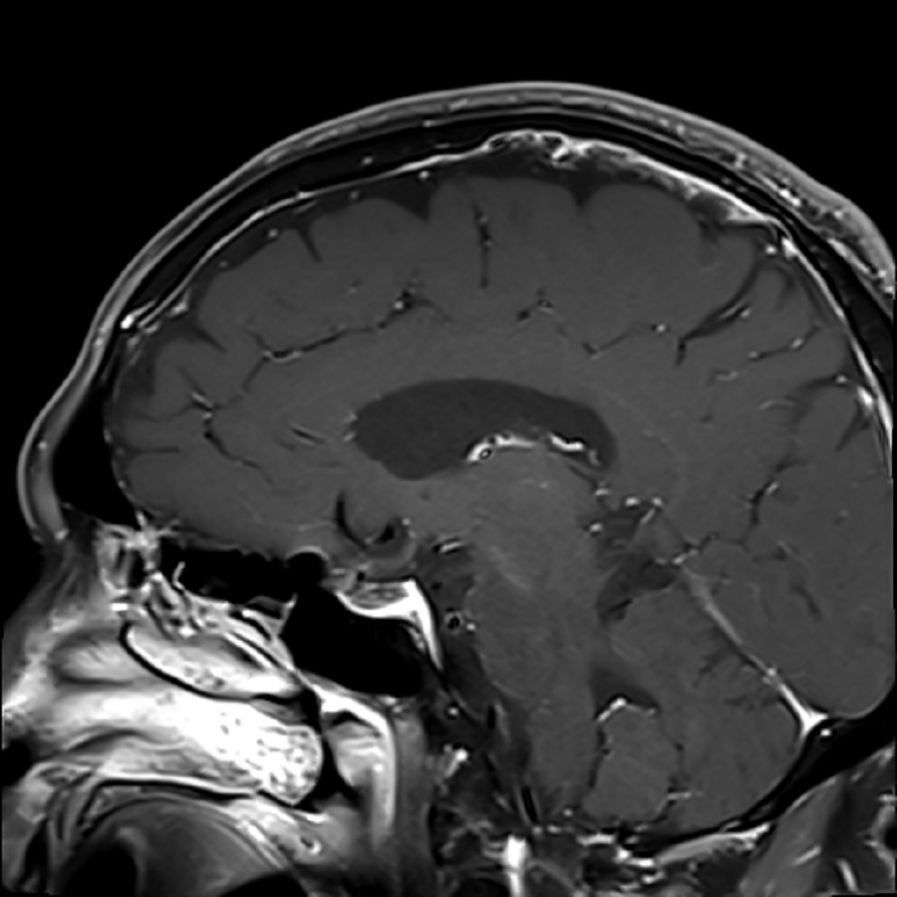
Follow-up MRI obtained approximately three and a half months following initial imaging T1 sagittal MRI showing complete resolution of the mass with no evidence of acute or chronic hemorrhage.

**Figure 5:**
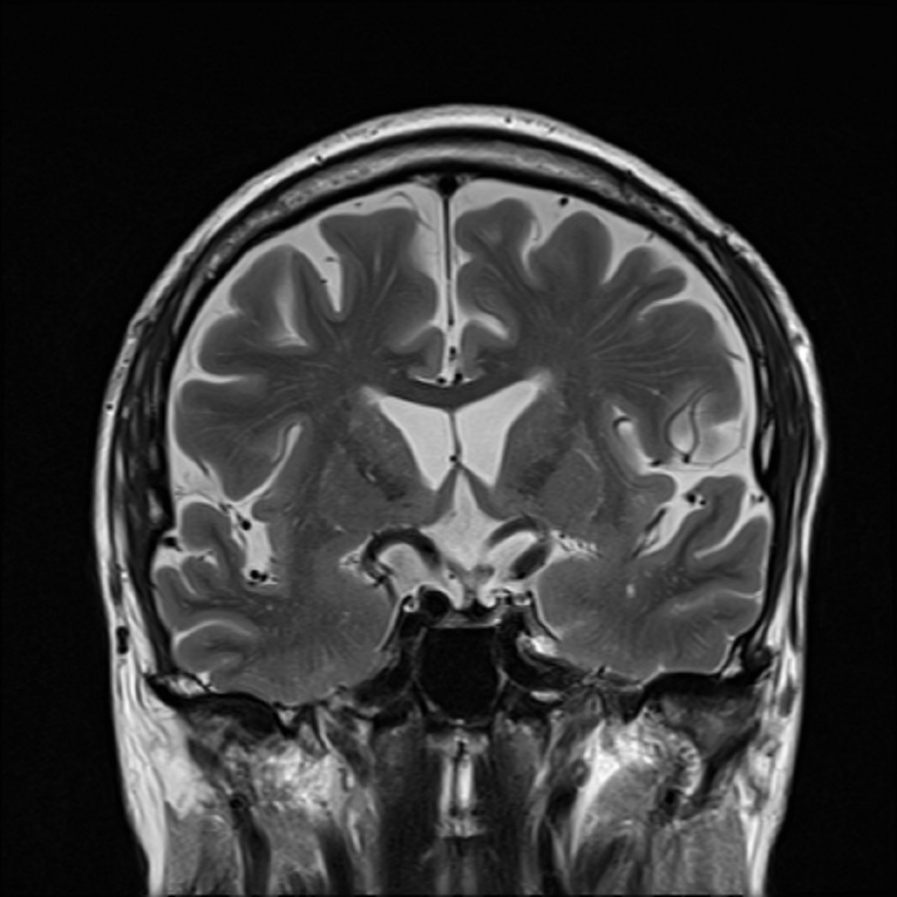
Follow-up MRI obtained approximately three and a half months following initial imaging T2 coronal MRI demonstrating complete resolution of the mass.

The patient was referred to our institution for outpatient evaluation of his sellar/suprasellar lesion. On neurologic examination, he was found to have a bitemporal hemianopsia; however, the remainder of his neurologic examination was nonfocal. Based on his outside laboratory values, MRI findings, and physical examination we believe the patient harbored a non-functioning pituitary macroadenoma. Given the degree of chiasmal compression and subjective visual field deficit, we felt that surgical resection via an endoscopic endonasal transsphenoidal approach was indicated. As is our protocol, a planning MRI and computed tomography (CT) scan of the brain and skull base were ordered and performed. The repeat imaging was done three and a half months after his initial MRI. The new MRI (Figures 3-5) showed that the sellar/suprasellar mass was no longer present and only normal enhancing pituitary tissue was noted along the sellar floor.

The infundibulum was now visible and normal in appearance. The optic chiasm had also normalized in position. There was no sign of acute or chronic blood products within the sella, and on further interview the patient denied any episodes of sudden onset of headache or change in vision.

## Discussion

We present a patient with an incidentally discovered NFPA, which spontaneously regressed over the course of approximately three and a half months. The patient had an MRI with imaging characteristics classic for a NFPA and documented visual field defects. A routine preoperative planning MRI demonstrated complete disappearance of the NFPA.

The differential for sellar and suprasellar lesions is broad and includes not only neoplastic entities such as pituitary adenoma, metastases, Rathke’s cleft cyst, craniopharyngioma, and meningioma but also vascular lesions and inflammatory lesions such as sarcoid and autoimmune hypophysitis. One must certainly consider this broad differential when evaluating this case. While the clinical, endocrinologic, and imaging characteristics supported a diagnosis of NFPA for our patient, there are a few specific alternative diagnoses that warrant discussion. The first of which is autoimmune hypophysitis. Autoimmune hypophysitis is a disorder with a strong temporal relationship to pregnancy where there is lymphocytic infiltration of the pituitary gland and infundibulum frequently leading to hypopituitarism, diabetes insipidus or hyperprolactinemia. Our patient did not match the classical clinical picture of autoimmune hypophysitis (young peripartum females presenting with pituitary dysfunction), although there are case reports in the literature of males and elderly patients presenting with autoimmune hypophysitis [3]. Additionally, our patient had a cystic component on MRI, which is radiographically inconsistent with autoimmune hypophysitis and he lacked enhancement of the anterior pituitary and stalk thickening which is thought to be specific for lymphocytic hypophysitis. The patient did, however, receive physiologic corticosteroid replacement during his initial hospitalization (20 mg of prednisone in AM and 10 mg of prednisone in PM), which could potentially have led to regression of lymphocytic hypophysitis.

Another possible diagnosis is pituitary apoplexy with interval resolution. Pituitary apoplexy is a clinical syndrome caused by sudden hemorrhage and/or pituitary infarction. It is rare, affecting only 2-12% of pituitary adenomas and is generally considered to be a neurologic emergency. The signs and symptoms can vary; however, headaches are present in greater than 80% of cases, visual disturbances and oculomotor palsies accompany greater than 50% of cases and resultant endocrine dysfunction is well documented [4].^ ^Interestingly, it has been described by Armstrong, et al. [5] as a potential cause leading to regression of pituitary adenomas. Nonetheless, given the denial of any acute symptoms by our patient in the time between the two MRI studies this seems to be less likely.

The natural history of non-functioning pituitary adenomas is not well studied because the majority of pituitary macroadenomas with chiasmal compression are treated surgically to prevent further vision loss. Research conducted by Igarashi, et al. [6] in Japan suggests that MRI characteristics of the tumor, specifically cystic nature, can be correlated with spontaneous decrease in size. A meta-analysis by Dekker, et al. suggests that approximately 10% of non-functioning pituitary macroadenomas show some degree of shrinkage with follow-up imaging, however only one reported case describing complete spontaneous regression of pituitary macroadenomas has been reported in the literature [7]. Bahar, et al. [8] discussed the case of a 46-year-old woman who presented with a pituitary macroadenoma that spontaneously regressed. Unlike our patient, whose NFPA was incidentally found due to confusion attributable to a congestive heart failure exacerbation, their patient presented with amenorrhea, weight loss, and malaise. Her macroadenoma, measuring 1.8 x 1.5 x 1.5 cm was also non-functioning; however, her endocrine workup revealed pan hypopituitarism whereas our patient had a normal endocrine workup. Finally, unlike our patient, she had no visual deficits. Despite these differences, the patient in the study by Bahar, et al. also had an NFPA with compression on the optic chiasm that completely spontaneously regressed by the three-month follow-up MRI with no signs of acute blood or pituitary apoplexy on this follow-up imaging.

## Conclusions

Further research into the natural history of NFPA could shed great insight into indications for and timing of surgical intervention for these non-secretory macroadenomas. Cases such as these suggest that imaging should be obtained as near the planned operative intervention as possible in order to minimize unnecessary operations and intraoperative complications.
